# Characteristics of Epstein–Barr virus reactivation after allogeneic haematopoietic stem cell transplantation in patients with chronic active Epstein–Barr virus disease: favorable responses to rituximab

**DOI:** 10.1038/s41409-020-01193-7

**Published:** 2021-01-08

**Authors:** Na Wei, Yini Wang, Jingshi Wang, Lin Wu, Zhao Wang

**Affiliations:** Department of Hematology, Beijing Friendship Hospital, Capital Medical University, Beijing, China

**Keywords:** Infectious diseases, Haematopoietic stem cells, Adverse effects

## To the Editor:

Epstein–Barr virus (EBV) belongs to the herpesvirus family. The infection rate exceeds 90% worldwide. Primary EBV infection is occult and lacks typical symptoms. The virus quickly enters the latent infection phase and is characterized by a lifelong presence in B cells [1]. In some cases, patients develop persistent fever, lymphadenopathy, hepatosplenomegaly, and significantly elevated EBV-DNA load and/or abnormal EBV antibodies in blood or EBV-encoded RNA and viral proteins in affected tissues [2, 3]. When these situations persist for more than 3 months, it is determined as chronic active Epstein–Barr virus infection (CAEBV). In Chinese patients, the most infected lymphocyte cell types during CAEBV infection are T and NK cells.

Without treatment, CAEBV patients develop progressive cellular and humoural immune deficiencies complicated by opportunistic infections, haemophagocytic lymphohistiocytosis (HLH), multiple organ failure, and lymphoma [4], often leading to death. The main purpose of CAEBV treatment is to clear EBV-infected cells and to avoid the occurrence of fatal complications. Unfortunately, the present treatment protocols, including traditional antiviral drugs, antitumor chemotherapy and immunotherapy, temporarily relieve symptoms without clearing EBV. Allogeneic haematopoietic stem cell transplantation (allo-HSCT) is the only effective method for curing CAEBV, but relapse and transplant-related mortality are still high and affect overall survival (OS) [4, 5].

EBV reactivation is a common complication post allo-HSCT. With the development of haploid, unrelated donor transplantation, and the application of antithymocyte globulin (ATG) in pretreatment, the incidence of EBV reactivation increases significantly. Some cases rapidly progress to posttransplant lymphoproliferative disease (PTLD), with a high mortality rate. CAEBV patients also have a risk of EBV reactivation post HSCT. However, unlike other blood disease patients, EBV DNAaemia post HSCT in CAEBV patients can be traditional B-cell EBV-PTLD; it can also be a result of CAEBV recurrence. This is the first study to analyse EBV-DNA changes in CAEBV post HSCT.

Thirty-two CAEBV patients who received allo-HSCT at the Hematology Department, Beijing Friendship Hospital, Capital Medical University from January 2018 to June 2019 were enrolled in the study. This retrospective analysis was approved by ethics committee of Beijing Friendship hospital. All patients met CAEBV diagnostic criteria [6]. The median age was 27 years (7–51 years). Males and females accounted for 62.5% and 37.5%, respectively. The median time from diagnosis to transplantation was 7 months (3–27 months). In addition, 71.9% of the patients had a history of HLH [7]. The EBV-infected lymphocyte types were T or NK cells or multiple lines. All patients received at least one cycle of etoposide-/dexamethasone-based chemotherapy pretransplant. Disease status pretransplant was defined as described previously [8]: there were five with no response, twenty-one in partial remission, and six in complete remission.

Donors were matched (15.6%) or haplo-identically (84.4%) related. Total body irradiation- and busulfan-based conditioning accounted for 59.4% and 40.6% of the patients, respectively. All patients used ATG during conditioning. Graft-vs.-host disease (GVHD) prevention was performed using cyclosporine in combination with mycophenolate mofetil and short-term application of methotrexate. No death during conditioning was recorded. The survival time after transplantation for all cases was more than 30 days.

Real-time quantitative PCR (RT-PCR) was used to detect EBV-DNA levels in peripheral blood mononuclear cells (PBMCs) and plasma (EBV-DNA quantitative fluorescence diagnosis kit, Hunan Shenghus Biological Technology Co., Ltd). EBV-DNA (in PBMCs and plasma) with an estimated <1000 copies/ml was defined as EBV negative. In patients who were EBV negative, EBV-DNA in PBMCs or plasma with equal or higher concentrations than 1000 copies/ml twice in succession was defined as EBV reactivation. After EBV reactivation, analysis of EBV-infected lymphocytes was performed by magnetic bead sorting combined with RT-PCR to detect EBV-DNA levels in CD4+, CD8+, CD19+, and CD56+ cells. According to the above criteria, 3 patients were EBV-DNA negative pretransplant, and 27 were EBV-DNA negative post HSCT. Two patients who had EBV-DNA were positive until 30 days post HSCT and dropped out of the study.

Until May 2020, of the 30 EBV-DNA-negative patients with a median follow-up of 412 days (32–764 days) after transplantation, 22, accounting for 73.3%, experienced EBV reactivation. Fourteen were infected with B cells; seven had infected T cells, NK cells, or multiple lines. One patient died of central nervous system (CNS) complications before the EBV-infected cell test. The median reactivation time was 30 days (15–178 days). We observed that in CAEBV patients, EBV in PBMCs often became negative earlier than in the plasma after transplantation. During reactivation, PBMCs typically became positive earlier than the plasma, with ~2^log^ higher. Thus, it was necessary to monitor PBMCs and plasma simultaneously.

Treatment was given according to the EBV-infected lymphocyte type. Fourteen patients had B-cell infection, and 2 became negative after a reduction in immunosuppression (RI). One patient was under observation because of aGVHD and died of intestinal perforation 2 weeks later. Eleven patients were treated with rituximab; 9 became negative, and 2 remained positive. One patient became negative after EBV-CTL infusion. Another patient progressed to multi-line infection and experienced CAEBV relapse. There were no confirmed cases of PTLD [9]. Seven patients with T, NK, or multi-line infection were given rapid RI. Three developed aGVHD, one became EBV negative, one stayed alive with cGVHD and asymptomatic low-level EBV copies, and one died of CNS relapse. Three patients showed rapidly increased EBV-DNA loads as the chimaeric rate decreased, and CAEBV relapsed. Two died of HLH. One was treated with the DEP regimen combined with DLI, and EBV became negative; after 4 months, EBV became positive again, and the patient died of HLH encephalopathy. One patient had a multi-line infection, but the EBV copies in B cells were 2^log^ higher than those in T and NK cells, and fever and lymphadenopathy were present. The chimaeric rate in this patient did not decrease; monoclonal B cells were found in the peripheral blood using flow cytometry. He was suspected to have PTLD and treated with the R-CHOP regimen, then EBV became negative.

**Fig. 1:**
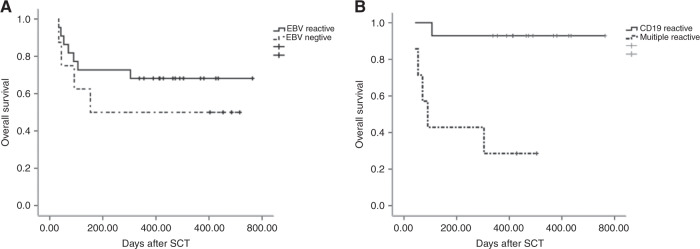
OS of different group patients. **A** OS comparing between the EBV reactivation and inactivation patients. **B** OS comparing between B cell infected and T, NK, or multi-line infected EBV reactivation patients.

Thirty-two patients had a median follow-up of 415 days (32–764 days) post HSCT until May 2020. Twenty-one survived, but 11 died. The 1-year OS rate was 66%. Death occurred in four cases of relapse and seven cases of non-relapse. No difference in OS between the EBV reactivation and inactivation groups (68.2 vs. 50%, *P* = 0.361) was detected (Fig. 1A). Among EBV reactivation patients, the OS of B cell-infected patients was significantly better than that of patients with T, NK or multi-line infection (92.9 vs. 28.6%, *P* = 0.001) (Fig. 1B).

The reported incidence of EBV DNAaemia post HSCT ranges between 0.1 and 63% according to different conditioning regimens, GVHD prevention, and EBV monitoring methods [9]. EBV-infected cells are usually B cells of donor origin [9]. In our study, the incidence of EBV reactivation was 73.3%, higher than that in past reports. Sixty-seven percent of the patients had infected B cells, whereas 33% had infected T cells, NK cells, or multiple lines. We considered that some patients had disease relapse rather than traditional EBV reactivation, which increased the overall reactivation, even though our patients were at high risk for B cell EBV-PTLD due to the use of ATG. Most PTLD occurred between the 2nd and 4th months post HSCT [10]. EBV DNAaemia was detected 1–2 weeks before PTLD [11]. The median EBV reactivation time among our patients was 30 days, earlier than previous reports. In subgroup analysis, the median reactivation time for infected B cells was 38 days; that for infected T cells, NK cells, or multiple lines was only 23 days. Relapse was more common in patients with early EBV reactivation. For such patients, we propose that EBV-DNA be monitored early and intensively.

Rituximab preemptive treatment was employed in high-risk patients and led to a reduction in PTLD mortality. In our study, rituximab was also beneficial for B cell-type EBV reactivation. Eighty-one percent of the patients became EBV negative, which was higher than that previously reported [12]. We consider that this may be related to the earlier application of rituximab used in proven B cell-infected patients. Although patients with infected T cells, NK cells, or multiple lines were treated with RI and/or DLI, most died of relapse. Therefore, we suggest that more effective treatments need to be investigated in the future.

In conclusion, EBV reactivation post HSCT in CAEBV is common; it may have a higher incidence and occur earlier than in other allo-HSCT patients. Thus, early and intense monitoring after transplantation are required. Similar to other transplant patients, EBV reactivation post HSCT in CAEBV mainly infects B cells, and rituximab is useful. The OS of multiple or non-B cell-type reactivation is poor, thus suggesting the strong need for the development of new treatments.

## Supplementary information

editing certificate
