# Supplementary material for: Characteristics of Epstein–Barr virus reactivation after allogeneic haematopoietic stem cell transplantation in patients with chronic active Epstein–Barr virus disease: favorable responses to rituximab
Source: Bone Marrow Transplant. 2021 Jan 8;56(6):1449–51. doi: 10.1038/s41409-020-01193-7 (PMC8189911; doi:10.1038/s41409-020-01193-7)
Supplement: Supplementary file 1 — editing certificate [file 41409_2020_1193_MOESM1_ESM.pdf]

SPRINGER NATURE

Author Services

Editing Certificate

This document certifies that the manuscript

Characterization of Epstein-Barr virus reactivation after allogeneic haematopoietic stem cell transplantation in chronic active Epstein-Barr virus disease: earlier and higher, rituximab is also useful

prepared by the authors

Na Wei, Yini Wang, Jingshi Wang, Lin Wu, Zhao Wang

was edited for proper English language, grammar, punctuation, spelling, and overall style by one or more of the highly qualified native English speaking editors at SNAS.

This certificate was issued on **November 21, 2020** and may be verified on the SNAS website using the verification code **D6AF-5154-D4C7-1BCB-3EB7**.

Neither the research content nor the authors' intentions were altered in any way during the editing process. Documents receiving this certification should be English-ready for publication; however, the author has the ability to accept or reject our suggestions and changes. To verify the final SNAS edited version, please visit our verification page at secure.authorservices.springernature.com/certificate/verify.
If you have any questions or concerns about this edited document, please contact SNAS at support@as.springernature.com.

SNAS provides a range of editing, translation, and manuscript services for researchers and publishers around the world.
For more information about our company, services, and partner discounts, please visit authorservices.springernature.com.
